# 

**Published:** 1858-10

**Authors:** 


*The Dental Convention.*—For the summary of the proceedings of the Dental Convention held at Cincinnati in August last, we are indebted to a professional friend who was present on the occasion; and for which kindness we beg to tender him our grateful acknowledgments. We regret that circumstances which we could not control prevented the senior editor from attending. The meeting, as will be seen from the report of the proceedings, was interesting. Not having a copy of the papers read on the occasion, we are unable to publish any of them in the present number of the Journal. We may copy some of them from other Journals in our next issue. The next meeting is to be held at Niagara Falls in August, 1859.

*The New York Dental Journal.*—The first number of this new candidate for professional patronage was issued on the first day of July last. The manner in which it is gotten up is creditable alike to the editors and publishers.

Obituary.—It is with regret that we announce the death of Dr. C. O. Cone, which occurred on the 1st of August last, as appears from the communication of the sad event to the American Dental Convention, then in session at Cincinnati, Ohio. The Doctor's health had been on the decline for nearly two years, and was so feeble the greater portion of this time as to prevent him from attending to the duties of his profession. He came to Baltimore about twelve years ago, and until within the last two, had devoted himself with untiring energy to his professional calling, and, as an operator, had acquired a high degree of excellence.

As our last form was about to be put to press, we were informed of the death of Dr. Elisha Townsend, of Philadelphia, but whether he died abroad or at home, we did not learn. As a dentist, Dr. T. ranked among the first in Philadelphia, and it is with sincere regret that we are called upon to announce his death.

In Philadelphia, on the 21st of July, 1858, Dr. R. T. Reynolds, in the 47th year of his age.

We learned with sincere sorrow, the sudden and unexpected death of Dr. Reynolds. He was a dentist in large practice, and was particularly celebrated for the beauty of his mechanical work. In this department he was *facile princeps.* He had a method of his own which was remarkably successful. He leaves behind him a family, and a large circle of friends and acquaintances to deplore his loss.

### INDEX.—VOL. VIII.

PAGE.

A.

Analysis of Salivary Calculus, 12
Amyline, . . . 17
Adapted Forceps, . 67
American Journal of Science and Arts, . . . 141
Anglo-American Dental Depot, 144
Address to the Graduating Class of Balt. Col. of Dental Surgery, Valedictory, . . 149
Allport, W. W., on Diseases of the Teeth, . . . 164
Artificial Teeth, on the Insertion of, . . . . . 49
Affections of the Upper Jaw, Statistics of, . . 147
Artificial Teeth, Fatal Pericarditis caused by Lodgment of, in Œsophagus, . . . 279
Artificial Teeth, Swallowing, 287
American Dental Convention, 289, 477, 585
Allport's Dental Ledger, . 291
Appointment, . . 292
Am. Medical Association, . 355
Association of Dental Surgeons, Mississippi Valley, . 382
Anæsthesia, Local, Electric, 433
Adhesive Gold Foil, . 443
Am. Dental Review, . . 444
Anæsthetic, another proposed, 584
American Dentists in Europe, 585

B.

Boston Society Medical Improvement, Extract from Records of the, . . . . 62, 124
Beware of Green Flounces, . 142
British and Foreign Medico-Chirurgical Review, . 146
Boot on the other leg, . 147

PAGE.

Baltimore Dental College, Commencement of, . . 290
Buccal Mucous Membrane, Gangrene of, caused by cold, 441
Baltimore College of Dental Surgery, . . . 584

C.

Coiled Gold for Filling Teeth, 3
Complete Luxation of Lower Jaw, . . . . 46
College of Dentists, Special Meeting of, . . . 78
Circulation in the Capillaries, 139
Congenital and Hereditary Transmissions of Diseases, . 194
Calculus, Salivary, . 231
Convention, Dental, . . 240
Convention of the N. Carolina State Dental Society, . 245
Chemistry among Dentists, 287
College, Dental, Baltimore, Commencement of, . . 290
Cartier, Alonzo L., M. D., on Pseudoplasmata, . 297
Contributions to Theoretical Dentistry, . . 331
College of Dentists, Monthly Meeting of, . . 390, 503
Cincinnati Lancet and Observer, 442
Charleston Medical Journal and Review, . . . 444
Convention, American Dental, 444, 585
Council of the College of Dentists, 583
Carpenter, Alfred, M. B., Lecture by, . . . 549
Chemistry, more Dental, . 581
Chancre on the Finger, Communicated by a Patient, . 583
Chemistry, Elements of Inorganic, . . . 586
Cone, C. O., Obituary Notice of, 588

PAGE.

D.

Deformity from Contraction of the Arch of Superior Maxillary and Irregularity of the teeth, successfully treated, . 56
Dalrymple, Dr. W., on Deformity from Contraction of the Arch of Superior Maxillary and Irregularity of the Teeth, successfully treated by, . . 56
Dixon, James B., on Prosopalgia and Tic-Douloureux, . 113
Disease of the Cavities of Molar Teeth in Horses, . 143
Dental Depot, Anglo-American, 144
Dentifrice, . . . 147
Dodge, J. Smith, Jr., M. D., Dental Histology, . . . 164
Dental Convention, . 240
Dental Convention, American, 289
Dictionary of Medical Science, 290
Dentures, Artificial, Lower, 337
Diathesis, Strumous, case of, 412
Dental Surgery, Lecture on, 549
Dental Chemistry, more, . 581

E.

Extraordinary Successive Development of Teeth, . 16
Extract from Records of the Boston Society for Medical Improvement, . . 62, 124
Exfoliation of the Sockets of two Teeth, . . . . 145
Erosion of the Teeth, . . 281
Eve, Paul F., Dr., Account of a Patient who Swallowed Artificial Teeth, . . . . 431
Excision of the Tongue, . 432
Electricity as an Anæsthetic, 433
Edmunds, Dr. G. H., Obituary of, . . . . 295

F.

Fehrlin, M. J., on Luxation of the Bicuspid Teeth, . 117
Fatal Pericarditis, caused by lodgment of Artificial Teeth in Œsophagus, . . 279
Forbes, Isaiah, M. D., Hemorrhage from the Superior Maxilla, . . . . . 405
Forceps, Elevator, . 431
Filling Teeth with Soft Sulphur, 441
Facial Nerve, Paralysis of, . 586

PAGE.

G.

Gedney, Richard S., Poetical Works of, . . 21
Guilbert, Dr., influence exerted upon the Teeth by Potable Waters, . . . 44
Greg, Dr. D. P., Letter from, on Insertion of Artificial Teeth, 49
Glands, Thyroid, . . 141
Green Flounces, beware of, 142
Gilman, Dr. John C., Obituary of, 148
Gill, Dr. S. L., Case of Strumous Diathesis, . . . 412
Gangrene of Buccal Mucous Membrane, caused by cold, 441
Gold Foil, Adhesive, . 443

H.

Histology, Nervous, . 137
Heart, Nerves in the, . . 138
Hemorrhage, Superior Maxilla, 405
Hypertrophy of the Tongue, 413
Hamilton, F., Tumor of the Parotid, . . . . 416
Heart Action, . . 442
Heart, Sounds of, . . 442
Hydrogen, . . . 462
Handy, Prof. W. R., Obituary, 293

I.

Influence exerted upon the Teeth by Potable Waters, . 44
Iris, Physiology of the, . 146
Irregularity of the Teeth, 407
Inorganic Chemistry, Elements of, . . . . 586
Illinois State Medical Society, 588

J.

Jaw, Lower, complete Luxation of, . . . . 46

K.

Knower, Charles, Fracture of the Inferior Maxilla, . 409

L.

Liver, Sugar in the, . 26
Lower Jaw, Tumors of, . 325
Lecture on the Principles and Practice of Dental Surgery, 549
Levison, Dr. J. L., Letter from, 579

Page.

M.

Memoir upon the Tumors of the Gums, . . . 38
Molar Teeth in Horses, Diseases of the Cavities of, . 143
Medico-Chirurgical Review, British and Foreign, . . 146
Minutes and Discussions of the New York Dental Association, 247
Microscopic and Comparative Anatomy of the Teeth, . 288
Medical Science, Dictionary of, 290
Medical and Surgical Journal, the Pacific, . . . . 291
Mississippi Valley Association of Dental Surgeons, . . 382
Maxilla, Inferior, Fracture of the, 409
Morong, C., Hypertrophy of the Tongue, . . . 412

N.

Nervous Histology, . 137
Nerves of the Heart, . . 138
New Forceps for Removing Superior Maxilla, . . 142
North Carolina State Dental Society, . . . . 245
New York Dental Society, Minutes and Discussions of, . 247
New Jersey Medical and Surgical Reporter, . . 443
New York Dental Journal, . 586
Nasmyth, Dr. R., Obituary Notice of, . . . 294

O.

Odontological Society, . 126
Obituaries, Handy, Professor Washington R., . 293
Nasmyth, Dr. Robert, 294
Edmunds, Dr. T. H., . 295
Somerby, Dr. R., . 296
Oxygen, by Prof. R. N. Wright, M. D., . . . 339
Obturation among the Ancient Greeks, . . . 441
Oglethorpe Medical and Surgical Journal, . . . 443
Osseous Union of Teeth, . 586

P.

Prosopalgia, Tic Doloreaux, 113
Perineuron, a New Element in Nerves, . . . 140
Physiology of the Iris, . 146

Page.

Piggot, A. Snowden, M. D., Valedictory Address, . 149
Pseudoplasmata, . 297, 444
Parotid, Tumor of, . 416
Paralysis of the Facial Nerve, 586

R.

Robinson, J., Adapted Forceps, 67
Royer, M. Jules, on Tumors of the Palate, . . . 120
Registers of the Human Voice, 135
Review of Dental Histology. 164
Russel, J. G., M. D., on Congenital Hereditary Transmission of Diseases, . . . 194
Researches on the Development of the Teeth, . . 575
Reynolds, Dr. R. T. Obituary, 588

S.

Supernumerary Teeth, . 53
Superior Maxillary, Deformity from Contraction of, and Irregularity of the Teeth, successfully Treated, . . 56
Society, Odontological, . 126
Superior Maxilla, New Forceps for Removing, . . 142
Statistics of Affections of the Upper Jaw, . . . 147
Saurel, Dr., on Tumors of the Gums, . . . 212, 445
Salivary Calculus, . . 231
Swallowing Artificial Teeth, 287, 424, 427
Severance, Alphonso, M. D., Extract from a Letter of, . 352
Sulphur, Soft, for Filling Teeth, 441
Somerby, Dr. R., . . 296

T.

Tumors of the Gums, Memoirs of the, . . . . 38
Teeth, Influence Exerted Upon the, by Potable Waters, . 44
Tumors of the Palate, . 120
Thyroid Glands, . . 141
Teeth, two, Exfoliation of the Sockets of, . . 145
Transposition of Teeth, . 145
Teeth, Wasting of the Roots of, 145
Teeth, Diseases of the, . 176
Tumors of the Gums, Memoir on the, . . 212, 445
Teeth, Erosion of, . 281
Teeth, Microscopic and Comparative Anatomy of the, . 288

Murray

| | Page. |
|---|---|
| Typographical Error, | 292 |
| Tumors of the Lower Jaw, | 325 |
| Teeth, Irregularity of, | 407 |
| Tongue, Excision of, | 432 |
| Teeth, Researches on the Development, | 575 |
| Teeth, Osseous Union of, | 586 |
| Townsend, Dr. Elisha, Obituary, | 588 |

V.

| | |
|---|---|
| Voice, Registers of the Human, | 135 |
| Valedictory Address, by A. Snowden Piggot, | 149 |

W.

| | Page. |
|---|---|
| Wasting of the Roots of Teeth, | 145 |
| Weight no Advantage in Artificial Lower Dentures, | 337 |
| Wright, Prof. R. N., on Hydrogen, | 462 |

Z.

| | |
|---|---|
| Zur Nedden, Contributions to Theoretical Dentistry, | 331 |

